# Preparation and physical characteristics of graphene ceramics

**DOI:** 10.1038/s41598-020-67977-5

**Published:** 2020-07-06

**Authors:** P. Głuchowski, R. Tomala, A. Jeżowski, D. Szewczyk, B. Macalik, I. Smolina, T. Kurzynowski, W. Stręk

**Affiliations:** 1 Institute for Low Temperature and Structure Research, Polish Academy of Sciences, 50422 Wroclaw, Poland; 2 Faculty of Mechanical Engineering, Wroclaw University of Science and Technology, 50370 Wroclaw, Poland

**Keywords:** Electronic properties and devices, Mechanical and structural properties and devices

## Abstract

Graphene, a two-dimensional structure of carbon, due to its structure has unique physico-chemical properties that can be used in numerous research and industry areas. Although this structure is already well known, there are still technological (and cost) barriers which do not allow to produce this material in large quantities and hence prevent its use in various applications. For this reason, many technologies are currently being developed to obtain graphene in forms that would enable its widespread use. The graphene-like ceramics were fabricated by the high isostatic pressure method at different temperatures. This technique allows to obtain dense ceramics with various shapes. The structure and morphology of sintered graphene were investigated by XRD, SEM and the Raman spectroscopy. The hardness, thermal conductivity and electric transport measurements recorded in a wide range of temperatures were used to analyze the physical properties of the obtained ceramics.

## Introduction

The term graphene appeared for the first time in 1987^1^ to describe a large 2D sheet of fused hexagonal rings of carbon atoms forming honeycomb layers. However, the first graphene flakes were obtained by Novoselov^2^ by exfoliation of highly oriented pyrolytic graphite (HOPG). This was done by pressing an adhesive tape onto the HOPG and then pulling it off; this leaves graphene in the adhesive tape. Subsequently, the adhesive strip is pressed onto a silicon wafer with a thin silicon dioxide layer and pulled off again. Thereafter, graphene becomes visible by suitable optical methods. This method is very time-consuming, and only very few, albeit high-value, samples are obtained. After this discovery, many new graphene preparation techniques such as epitaxy^3,4^, reduction from SiC^5^, reduction of graphite oxide^6,7^ carbon nanotube slicing^8^ or solvent exfoliation^9^ were developed.

Graphene demonstrates a series of exceptional physical, chemical and mechanical properties. It has a large theoretical specific surface area (2,630 m^2^ g^−1^), high intrinsic mobility (200 000 cm^2^ v^−1^ s^−1^)^10^, high Young’s modulus (∼ 1.1 to 2.0 TPa)^11^, thermal conductivity (up to 4,300 Wm^−1^ K^−1^)^12^, optical transmittance (up to 98%)^13^, and good electrical conductivity^14,15^. The combination of these unique properties makes graphene an excellent candidate for many potential applications as supercapacitors^16,17^, ultrafast transistors^18^, biosensors^19,20^, chemical sensor^21^, light source^22^, photocatalysts^23–25^ or medium improving mechanical properties^26^. In all these applications graphene is used in different forms, such as polymer-based composites^27,28^, coloids^29^, foam^30^, thin films^31,32^ or ceramics^33,34^.

The conventional sintering technique, such as Hot Pressing (HP) and Hot Isostatic Pressing (HIP) allows sintering of ceramics with relatively long processing times and high temperature. Both factors result in grain growth and simultaneous degradation of graphene in ceramic composites. In order to avoid these problems, novel sintering techniques for ceramics, such as spark plasma sintering (SPS) or microwave sintering are being exploited. These methods allow to lower the sintering temperature and shorten the dwell times. Well consolidated ceramic composites in the form of plates prepared by SPS were reported by Walker et al.^35^. This method used a homogenous mixture of graphene plates and silicon nitride particles densified at 1,650 °C. Another challenge associated with preparing graphene ceramic composites is to develop processing routes that produce a good dispersion of graphene in the ceramic matrix. Despite intensive research in this area, as yet nobody has developed methods for sintering pure graphene ceramics.

The paper describes a new method of obtaining tough graphene ceramics without any additives. Sintering is based on high pressure (up to 5 GPa) and relatively low temperature (from 500 °C). The high pressure applied during sintering arranges graphene flakes parallel to each other and does not allow for grain growth, which results in keeping the structure and properties of the graphene in the ceramics. Since the pellet is closed in a hermetic container only a small part of graphene is oxidized. The advantage of obtaining graphene ceramics by high pressure sintering is the possibility of using any type of graphene (or graphene oxide). Additional sintering of graphene flakes does not require its further chemical or physical treatment. The high pressure sintering method allows for a simple formation of the composites by introducing additional compounds into the graphene powder. In addition, it should be emphasized that the method of obtaining ceramics in this way is relatively inexpensive and very fast. The development of this technology will allow to produce graphene ceramics as well as composites with designed unique physical properties, such as efficient persistent conductivity with a possibility of application in photovoltaic cells^36^. The prepared ceramics were characterized by XRD, SEM, Raman, thermal and electric measurements. It was found that sintering temperature and pressure have great impact on the thermal and electrical properties of the graphene ceramic.

## Experimental

The preparation of graphene ceramics was performed using the high isostatic pressure (HIP) technique as described elsewhere^37^. For sintering commercial available graphene flakes were used (GraphenX Synthetic Graphene by Graphene Technologies, Cheap Tubes Inc.). Before sintering, the pellets (10 mm in diameter and 2 mm in thickness) were formed by cold pressing under 0.2 GPa (called green body—GB). The sintering process was carried out in a high-pressure container. The samples of graphene ceramics were prepared by hot-pressing up to 5 GPa and temperatures in the range from 500 to 1,500 °C during 1 min.

The crystal structures, phase purities and morphology of the ceramics were analyzed with the X-ray diffraction (XRD) and electron microscopy (TEM/SEM) measurements. The XRD patterns were collected at room temperature between 10 and 60 degrees (in 2θ) by an X’PERT PRO PANalytical diffractometer using CuKα_1_ radiation (1.5406 Å, step: 0.03°). TEM images were made using Tesla BC 500 (90 kV, resolution 1.0 nm). SEM images were made with a FE-SEM microscope (FEI NovaNanoSEM 230). The Raman spectra were collected using Renishaw inVia Raman Microscope equipped with an IR laser (830 nm) and CCD camera under ambient conditions. The Raman wavelength shift ranged from 1,000 to 2,500 cm^−1^ and the spectral resolution was 0.7 cm^−1^. The laser spot size was 1.5 μm in diameter with × 50 magnification of the objective.

The hardness of the ceramic was characterized using a micro- and nano-hardness tester. Microhardness was checked using a Vickers indenter (Zwick Roel) with 25 g loading. Nanohardness was checked using a Berkovich indenter (Nanoindentation Tester Anton Paar NHT3).

The thermal measurements were carried out by the axial stationary heat flow method^38^. The experiment was conducted in the temperature range 4–300 K. Samples were prepared in a cuboid-like shape in order to provide heat flow through the same area along the sample and simultaneously minimize the geometrical error. The temperature gradient along the sample was induced by a small resistive heater glued to the top of a specimen and it was determined by means of a differential thermocouple, typically it was kept between 0.1 and 0.2 K. The measurement chamber temperature estimated by germanium and platinum thermometers was stabilized at the level higher than ± 3 mK. The temperature of the sample was measured by a constantan–manganin thermocouple. To avoid heat transfer between the sample and the environment, the measurements were performed under high vacuum conditions and four shields were mounted around the sample to reduce the heat losses due to radiation at the finite temperature^39^. The maximum experimental systematic error was below 15% (caused mainly by the uncertainty of sample geometry) and the spurious errors estimated from the point scattering did not exceed ± 2%.

The electrical impedance was measured employing an Alpha analyzer (Novocontrol GmbH) with an active sample cell operating in the frequency range 10^−2^–10^6^ Hz at room temperature with an ac voltage amplitude of 0.1 V^40^. The studied sample was placed between golden electrodes. The dc resistivity was examined by the four-contact method^41^. The thermoelectric power was determined in the temperature range 7–300 K by a steady-state mode using a semiautomatic instrument fitted into the transport liquid-helium Dewar^42^.

## Results and discussion

### Structure and morphology

**Figure 1:**
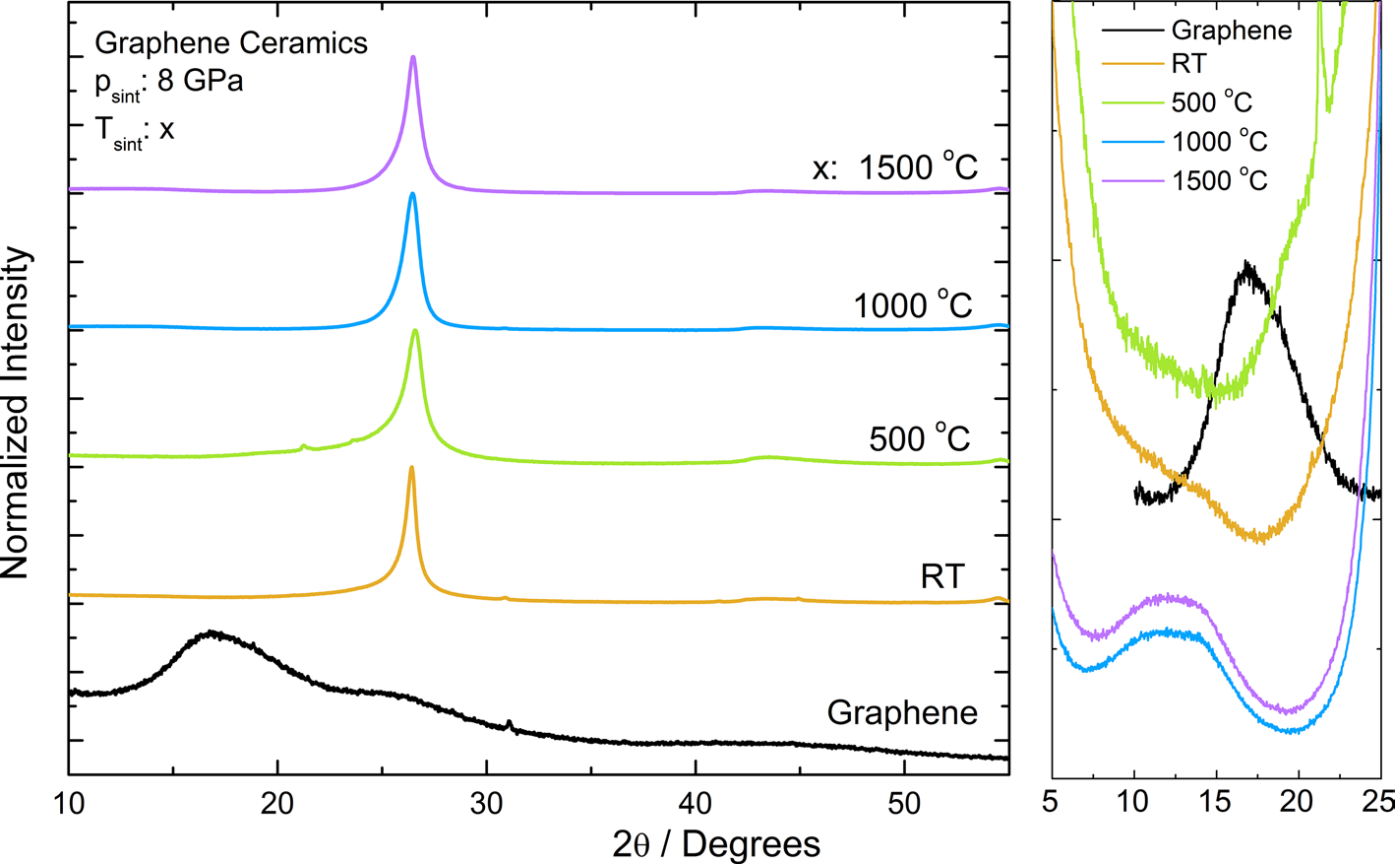
XRD diffraction pattern of graphene flakes, “cold pressed” pellet and ceramics sintered at different temperatures. On the left side magnified patterns in the range where diffraction reflection of the GO is observed are visible.

The sintered ceramics show no decomposition since only reflections resulting from graphene and graphite structure (Fig. 1) are observed. For ceramics sintered at the highest temperature only low intensity band is observed at about 10 – 15° coming from graphene oxide (GO). It is probably related to the appearance of oxygen on the surface of graphene flakes that are trapped between layers and during sintering at higher temperatures react with graphene. In the graphene flakes patterns, one can observe the broad band from 15° to 25° associated with the X-ray reflections of the graphene as well as the high peak at 26° observed due to graphite structure. After applying higher sintering temperature a new band about 10°–15° is observed, which indicates that GO appears in the sample. At 500 °C it is also observed that the intensity of the broad band at 15°–25° strongly decreases. Hae-Mi Ju et al.^43^ observed that with an increasing temperature reduction of graphene oxide to graphene, the peak 2Θ ≈ 26° was shifted to a higher degree, which was related to reducing distance d_002_ caused by removing intercalated water molecules and the oxide groups of hydroxyl and carboxyl groups. In the cold pressed pellets, the position of the peak is around 26.4°, and after sintering at 500 °C it was shifted to 26.6°, which indicates that the application of pressure results in decreasing interatomic distance. This behavior, combined with the fact that with increasing the sintering temperature the band observed at 26° becomes narrower, suggests a decrease in the strains in the ceramic. The average sizes of flakes (thickness) were determined by means of the Scherrer equation^44^ and was found to be about 14 nm for ceramics and 35 nm for graphene flakes. The calculations were made for the peak observed at 26° for ceramics and for the band about 18° for the flakes. The derivation of the Scherrer equation was given for particles with a spherical shape and it is only an approximation, however, the analysis of the XRD patterns showed that applying the pressure and increasing the sintering temperature results in the formation of a multilayered structure with higher density.

**Figure 2:**
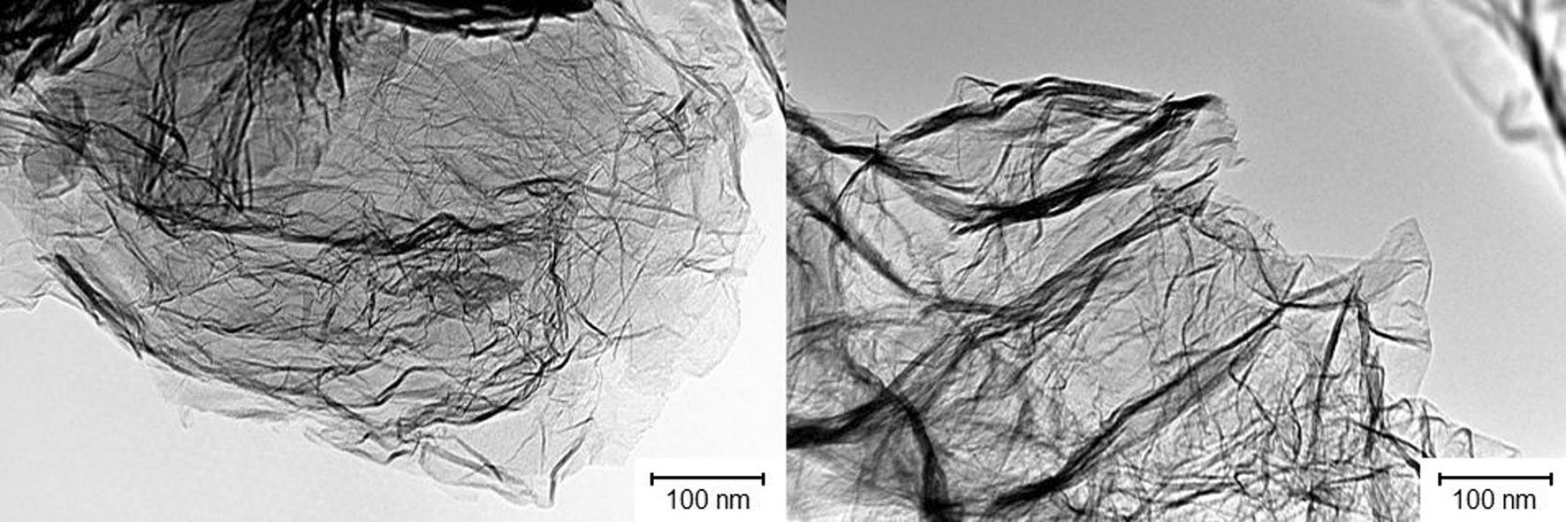
TEM images of graphene flakes.

To evaluate the surface morphology of the starting graphene flakes, TEM images were taken (Fig. 2). It is shown that single graphene flakes have a diameter of about 500 nm. It can be also observed that in some parts of the sheets, the multilayered structure appears what may be related to the “graphite” peaks observed in the XRD.

**Figure 3:**
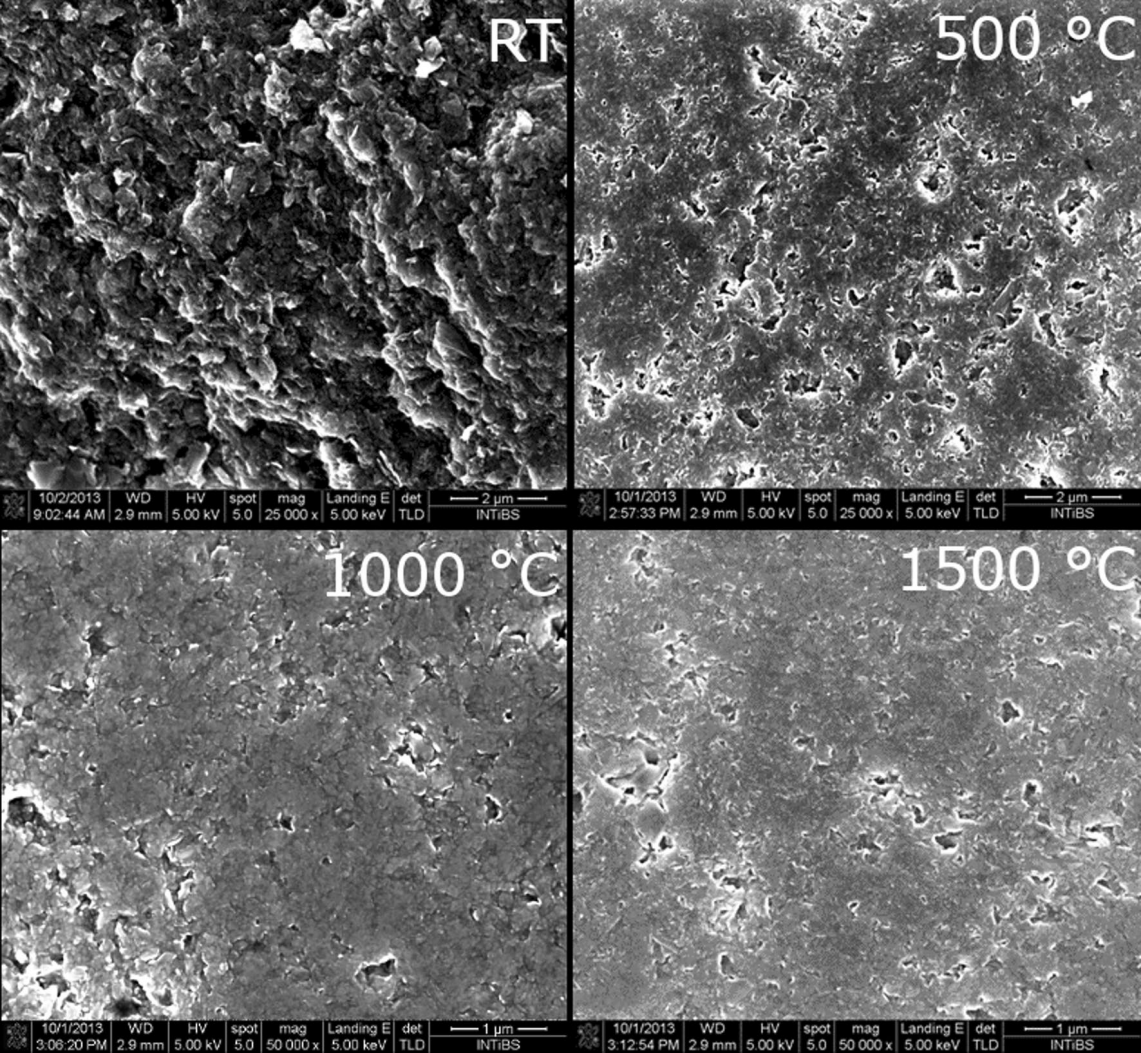
SEM images of graphene “cold pressed” pellets and ceramics sintered at different temperatures.

The SEM images of graphene GB and ceramics show their surface after polishing (Fig. 3). What should be noted is the difference between cold pressed (GB) and sintered ceramic. After pressing, the number of pores and their sizes significantly decrease, ceramics become consolidated and their surface is much smoother.

**Figure 4:**
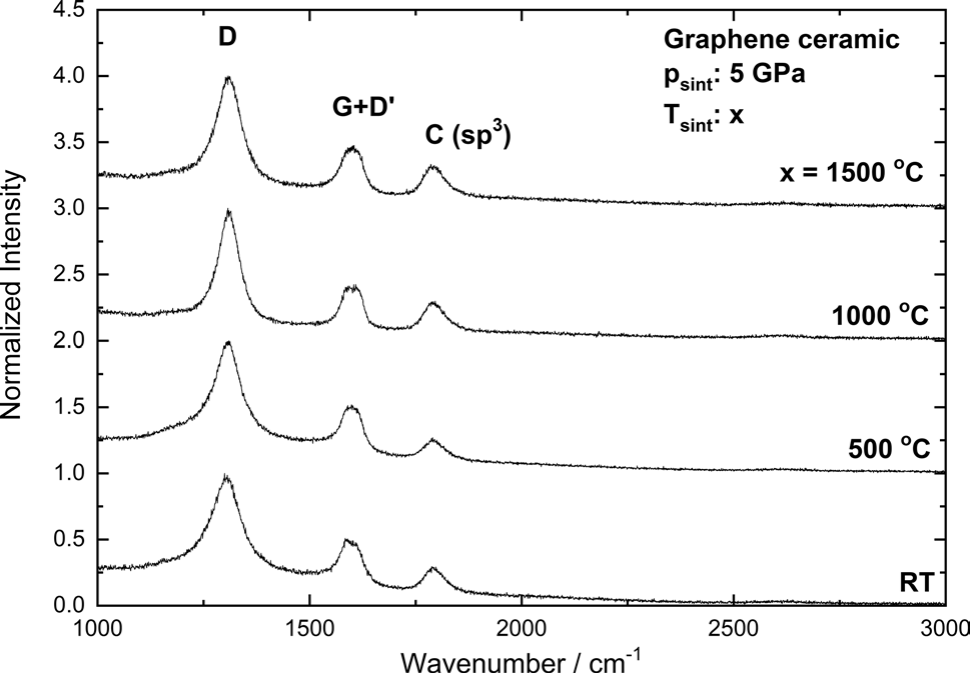
High-frequency first-order micro-Raman spectra of graphene “cold pressed” pellets and ceramics sintered at different temperatures.

The Raman spectrum of cold pressed pellet and graphene ceramics is composed of three broad bands (Fig. 4). The D-mode, appears at approximately 1,305 cm^−1^^45^, and the G-mode together with the D’-mode appears at approximately 1,590 cm^−1^^46^. At 2,600 cm^−1^ a very weak signal from the 2D-mode can be observed. The low intensity of this peak is caused by the low sensitivity of the CCD camera in this region. In the Raman spectra obtained from the samples with a small crystallite size (less than 0.5 μm, i.e. smaller than the wavelength of light), the presence of an additional dispersive peak centered at approximately 1,350 cm^−1^ is observed. This feature is assigned to the breathing of the carbon hexagons that become Raman active at the borders of the crystallite areas owing to the loss of translational symmetry^47^. The high intensity of the peak at 1,790 cm^−1^ indicates high fraction of tetrahedrally coordinated carbon (sp^3^ hybridization)^48^. Peaks in the range 1,700–2000 cm^-1^ could also be the result of the appearance of linear carbon chains^49^.

The peak at approximately 1,350 cm^−1^ is known as the disorder band or the defect band (D band). This band appears when sp^2^ carbon rings interact with the graphene edge or network defects. If the structure has a lot of defects (or graphene), the intensity of this band increases. The G band observed around 1,590 cm^−1^ comes from the C–C bond in graphitic materials, and is common to all sp^2^ carbon systems. It was shown that the shift of this band toward higher frequencies indicates the number of graphene layers. The intensity ratio of these two bands (I_D_/I_G_) may be used to quantify the disorder of the examined structures, because for bigger grains, or a multilayer graphene structure, the sp^2^ carbon hexagonal structure starts to disappear. The I_D_/I_G_ ratio is correlated with crystallite size (d) and is equal to the A/d ratio, where A is a constant for a fixed laser power^50^.

**Figure 5:**
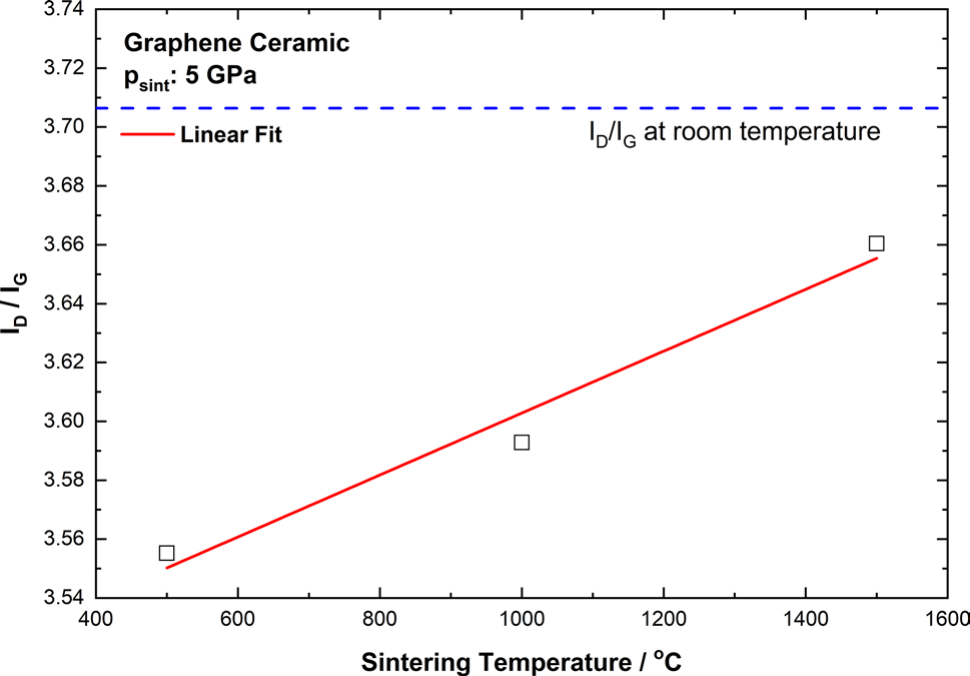
Intensity ratio of D and G bands calculated for ceramics sintered at different temperatures.

In our case the disorder in the ceramics increases with sintering temperatures (Fig. 5). This behavior contradicts the observations of Zhang et al.^51^ and Chen et al.^52^, made for a compressed graphene foil. They showed that by increasing the sintering temperature, the disorder factor decreases. This behaviour is probably related to two facts. First, both groups used the hot pressing techniques, but at a relatively low pressure (40 MPa and 29.4 MPa, respectively). In our case, the applied pressure reached 5 GPa (one order higher), which could introduce additional defects in the structure, and prevent the release of oxygen between the layers. The second difference compared to the presented publications is the fact that in our case the synthesis was not carried out under vacuum. Therefore, oxygen was not removed from the reaction atmosphere and the surface of graphene flakes. The oxygen trapped between the graphene flakes could additionally react with them, especially due to the fact that the synthesis conditions were extreme (very high pressure and temperature). The above facts are the reason for the increase in the disorder factor in ceramics with the growth of the sintering temperature. The high value of the intensities ratio calculated for pellets, obtained at room temperature, is related with a very small particle size in starting materials and high disorder in separated graphene sheets. After sintering at 500 °C, some graphene sheets are combined to form bigger particles, which could be observed in the SEM images.

### Hardness of the ceramics

**Figure 6:**
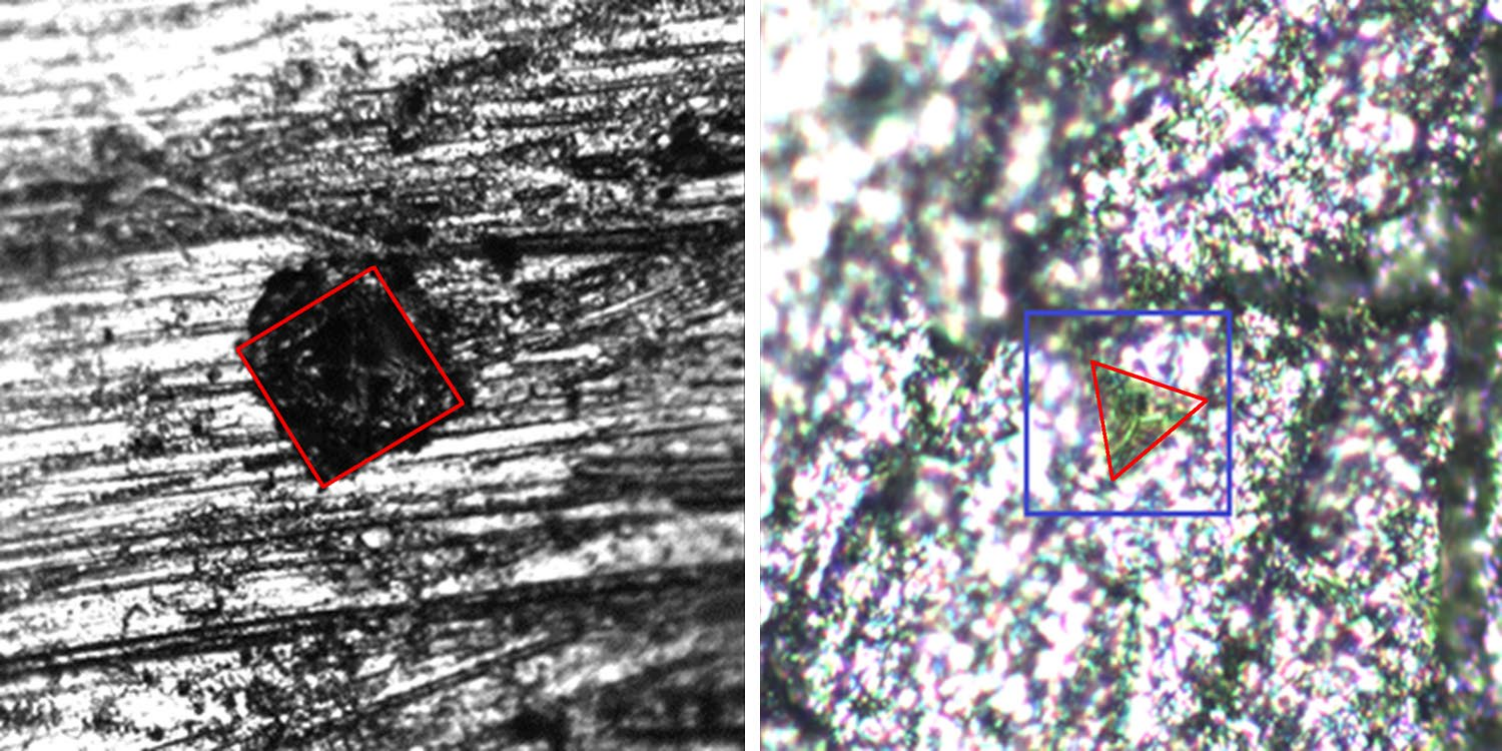
Trace on the graphene ceramic after applying force with Vickers microindenter (left) and Berkovich nanoindenter (right).

For the ceramics prepared at different temperatures, hardness was measured using two methods. For every sample, several measurements were made at different points on the surface to obtain the real hardness of each ceramic. For all samples hardness was checked using a nano- and micro- indenter (Fig. 6). Before the measurements all samples were polished using high grade grinding paper and as the last step also on paper for waveguide polishing, to obtain as smooth a surface as possible. Since the measurement with the nanoindenter imposes high requirements to the surface condition, the results have a higher error than in the case of the microindenter. Nevertheless, regardless of the measurement method used, there is a noticeable trend that shows an increase in hardness with rising sintering temperature (Table 1). The indentation tests on ceramics were systematically analyzed with Berkovich triangular and Vickers quadrangular pyramid diamond indenters. Figure 6 shows a typical footprint of a Vickers microhardness indenter measurement (a) and a Berkovich nanoindenter (b). In the case of the Vickers intender 25 g loading was used and the nanointender force was up to 2 mN. In the image of the ceramic surface, the marked area is the outline of the imprints which were used to calculate the hardness of the ceramics.

**Table 1:** Hardness measurement results for ceramics sintered at different temperatures.

	500 °C			1,000 °C			1,500 °C		
	HV0.025	Nanoindenter^b^		HV0.025	Nanoindenter^b^		HV0.025	Nanoindenter^b^	
		MPa	HV^a^		MPa	HV		MPa	HV
Average value	14.4	35	3	14	11	1	18.6	44	4.2
Error	2.1	–		1.4	–	–	2.1	18.2	1.9

^a^Calculated value.

^b^Force up to 2 mN.

### Thermal conductivity

**Figure 7:**
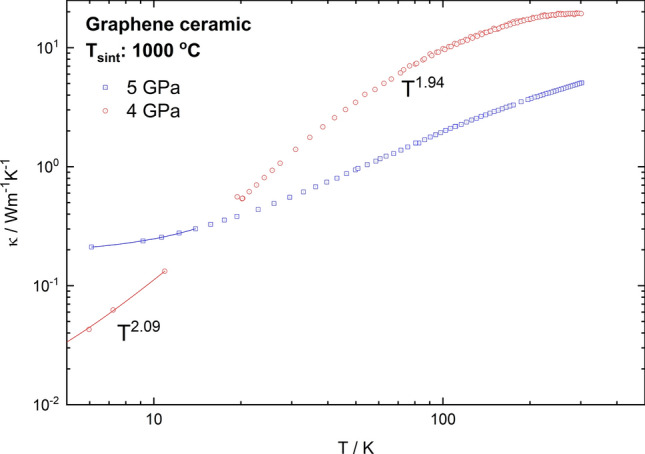
Temperature dependence of thermal conductivity of graphene ceramics obtained at 4 and 5 GPa.

The thermal conductivity of the graphene ceramic changed for samples obtained at different sintering pressures (Fig. 7). The results of thermal conductivity measurements are displayed on a double logarithmic scale. In the lowest temperatures, the thermal conductivity follows the T^2^ dependency, which is characteristic for an amorphous material^53^. This tendency is caused by some additional scattering mechanisms and may be explained by a closer observation of the structure of the investigated samples. The samples are significantly porous, and the investigations on such materials at low temperatures indicated that phonons are predominantly scattered by pores^54^. With increasing temperature, the conductivity of graphene samples grows at different rates—gently for the samples pressed with 5 GPa, and more firmly for the 4 GPa ones. For a high temperature range with increasing pressure, there is an unusual decrease in the value of thermal conductivity. It may be connected with the porosity of the samples or their quality. Consequently, in further research it would be recommended to carry out experiments with higher diversity in used pressure to see if maybe the influence of the sample origin on this particular case was not observed.

### Electric transport

**Figure 8:**
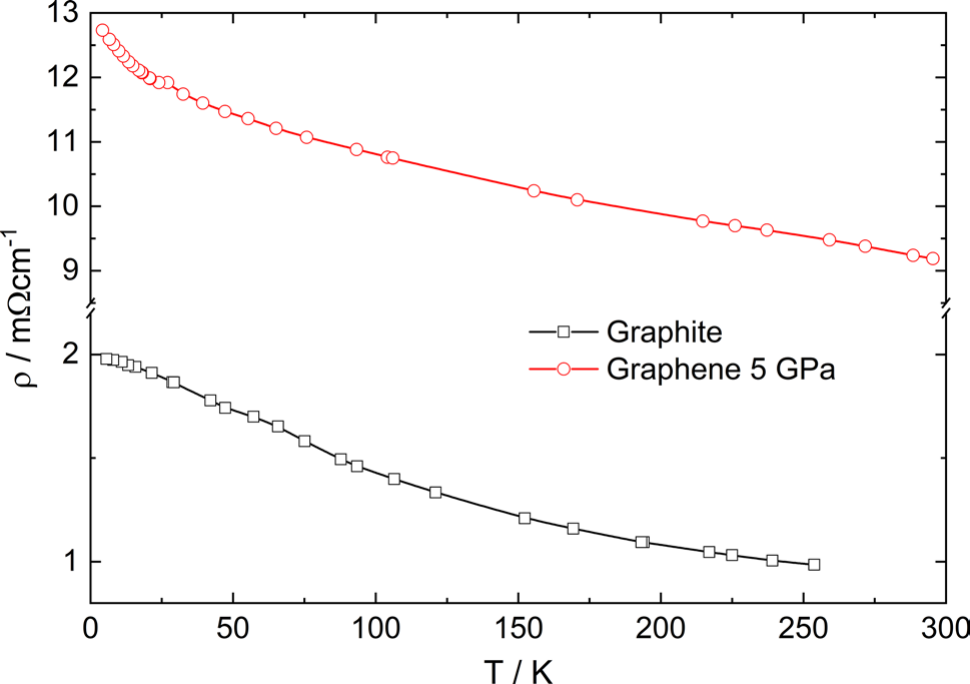
Resistivity of graphite and graphene ceramic sintered at 5 GPa as a function of temperature.

Within electric transport investigations, high purity graphite and graphene ceramics obtained at 5 GPa were studied by a four-contact method in the temperature range 2–300 K (Fig. 8). As can be seen, the values of graphene ceramic resistivity are an order higher than those of graphite in the whole temperature range and the temperature dependencies of both of the samples are different. While the graphite sample showed the semimetallic-like behavior, the graphene ceramic revealed clear semiconductor character of resistivity. The residual resistivity shows the semiconductor-like dependence of resistivity; the resistivity of the graphite sample skewing in the different way, which reveals some saturation at the temperatures below 10 K. This may indicate the semimetallic-like behavior of graphite sample which remains in agreement with the results obtained by García et al.^55^.

The extrapolation of resistivity in the high temperature region allows to determine the band gap of graphene ceramics. The band gap was obtained from the fitting of the temperature dependence of resistivity (see Fig. 8) by the application of the following relation:

$$\rho \left(T\right)={\rho }_{0}\mathrm{e}\mathrm{x}\mathrm{p}\left(\frac{{E}_{g}}{{k}_{B}T}\right)$$

where *E*_g_ is a band gap energy and *k*_B_—Boltzmann constant, *T*—temperature and *ρ*_0_ = 8 mΩ·cm. The fitting showed that the energy gap of graphene ceramic is smaller than thermal energy *k*_B_*T* in the whole temperature range and only near 40 K both of the values become similar. The calculated *E*_*g*_ reaches up to 7.6 meV at 237 K, whereas *k*_B_*T* = 20 meV at this temperature. Thus, it could be concluded that despite the semiconductor-like character of resistivity, the activation energy of graphene ceramic is rather smaller than *k*_B_*T,* which indicates the semimetallic-like dependency of this sample in the whole temperature range.

### Thermopower

**Figure 9:**
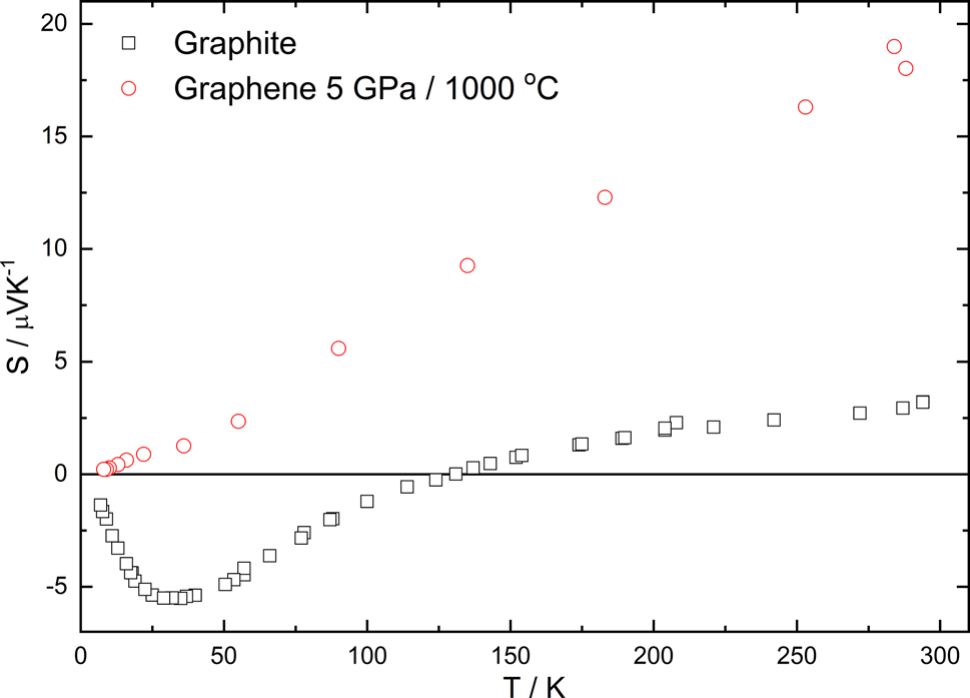
Thermopower as a function of temperature of graphite and graphene.

For both graphite and graphene ceramics obtained at 5 GPa and 1,000 °C, the temperature dependence of thermopower was measured (Fig. 9). It can be observed, however, that the dependencies are different. The graphite sample revealed semimetallic-like behavior with a minimum near 30 K. Interestingly, the values of thermopower changed the sign near 150 K indicating a change of the types of carriers from holes (above 150 K) to electrons (below 150 K). The graphene sample showed a typical semiconductor-like dependence of thermopower with a small deviation near 30 K. This is probably related with the phonon drag effect observed for the graphite sample at the similar temperature. By definition, the phonon drag effects appear at the temperature of *Ɵ*_D_/5, where *Ɵ*_D_ is the Debye temperature. It follows from this relation that *Ɵ*_D_ can be estimated for both of the samples. Thus, *Ɵ*_D_ = 150 K for the graphite sample and *Ɵ*_D_ = 100 K for the graphene ceramic. These values are rather smaller than those obtained for graphite and graphene in different studies with *Ɵ*_D_ = 402 K and *Ɵ*_D_ = 2,100 K for graphite and graphene, respectively^56,57^. The thermopower value of graphene ceramic at 300 K is *S* = 20 μV K^−1^. However, the thermopower of single, double and even more layered graphene at 300 K varies in the range from 6 μV K^−1^^58^ to 180 μV K^−1^^59^, depending on the measurement method. Hence, it is difficult to compare them but one can conclude that this value is higher than that of pure graphite.

It should be noted that the graphene ceramic obtained at 5 GPa and 1,000 °C revealed S = 22 μV K^−1^ at 300 K. This suggests a weak influence of the sintering conditions on the thermoelectric properties of graphene.

### AC conductivity

**Figure 10:**
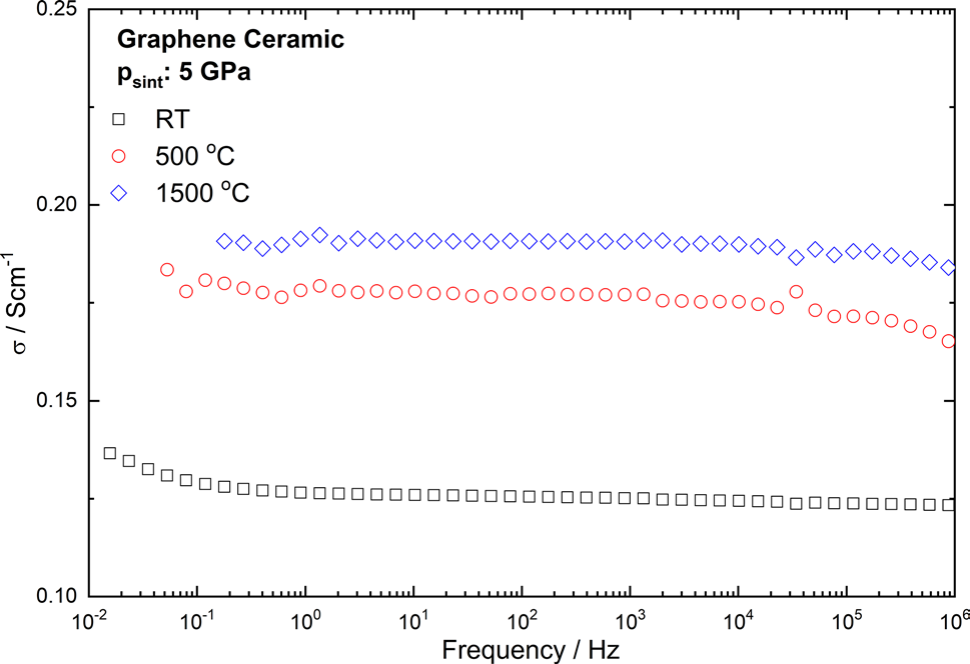
AC conductivity of graphene ceramics obtained in various conditions as a function of frequency.

The frequency dependence of the ac conductivity of graphene ceramics obtained at 5 GPa and room temperature, 500 °C and 1,500 °C are shown in Fig. 10. The measurements were performed along the height of the samples which is collinear with the direction of the applied pressure during the sintering. This can be interpreted as the response of c-axis conductivity, i.e. between graphene layers, according to SEM studies. All of the samples show similar linear dependencies of conductivity, which indicates resistor-like behavior without any relaxations in this frequency range. The conductivity of the samples obtained at 500 and 1,500 °C weakly decreasing at frequencies above 10 kHz, suggests the occurrence of the skin-effect. The main effect of sintering conditions is the rising of sample conductivity with an increase in sintering temperatures. Most probably, this relates to the formation of good electrical contacts between graphene plates. Taking into account that frequency dependence of the ac conductivity shown in Fig. 10 is mainly determined by the conductivity in the c-axis of graphene, it could be concluded that a rise in temperature may increase the interconnections between graphene layers and conductivity in this direction, respectively.

## Conclusions

In the paper the preparation method of pure graphene ceramic was presented for the first time. It was shown that in some cases even ceramics may be treated as HOPG (Highly Oriented Pyrolytic Graphite) and that their physical properties are more likely to be similar to graphene materials. It was also demonstrated that at high pressure and suitably selected sintering temperature, it is possible to obtain graphene-like ceramics with semiconductor properties. An increase in the sintering temperature leads to the densification of the ceramics and the growth of thermopower or AC conductivity. The presented method of graphene ceramic preparation may be utilized in the production of various types of sensors or transistors. Thanks to the simple production method, it is possible to manufacture this type of ceramics with admixtures of other ions or compounds, which can greatly extend the application areas of this material and can significantly improve its physical properties.
